# Extract Nutritional Information from Bilingual Food Labels Using Large Language Models

**DOI:** 10.3390/jimaging11080271

**Published:** 2025-08-13

**Authors:** Fatmah Y. Assiri, Mohammad D. Alahmadi, Mohammed A. Almuashi, Ayidh M. Almansour

**Affiliations:** 1 Software Engineering Department, College of Computer Science and Engineering, University of Jeddah, Jeddah 21493, Saudi Arabia; 2 Computer Science and Artificial Intelligence Department, College of Computer Science and Engineering, University of Jeddah, Jeddah 21493, Saudi Arabia; mmuashi@uj.edu.sa; 3 Saudi Food and Drug Authority (SFDA), Riyadh 11671, Saudi Arabia; amamansour@sfda.gov.sa

**Keywords:** LLM (large language model), OCR (optical character recognition), computer vision (CV) text extraction, nutrition label

## Abstract

Food product labels serve as a critical source of information, providing details about nutritional content, ingredients, and health implications. These labels enable Food and Drug Authorities (FDA) to ensure compliance and take necessary health-related and logistics actions. Additionally, product labels are essential for online grocery stores to offer reliable nutrition facts and empower customers to make informed dietary decisions. Unfortunately, product labels are typically available in image formats, requiring organizations and online stores to manually transcribe them—a process that is not only time-consuming but also highly prone to human error, especially with multilingual labels that add complexity to the task. Our study investigates the challenges and effectiveness of leveraging large language models (LLMs) to extract nutritional elements and values from multilingual food product labels, with a specific focus on Arabic and English. A comprehensive empirical analysis was conducted using a manually curated dataset of 294 food product labels, comprising 588 transcribed nutritional elements and values in both languages, which served as the ground truth for evaluation. The findings reveal that while LLMs performed better in extracting English elements and values compared to Arabic, our post-processing techniques significantly enhanced their accuracy, with GPT-4o outperforming GPT-4V and Gemini.

## 1. Introduction

Food product labels play a crucial role in the food industry by enabling compliance with food and health regulations and supporting online grocery stores in providing reliable nutritional information. These labels offer critical details, including ingredients, nutritional facts, serving size, and expiration dates, which consumers increasingly rely on to make informed dietary decisions. Recognizing food ingredients is essential for maintaining a healthy lifestyle as food labels serve as a bridge to reduce the information gap between food manufacturers and consumers [1]. Furthermore, food product labels play a critical role in identifying the detailing nutritional elements and values [2,3], monitoring expiration dates [4,5], and detecting halal status of products [6,7].

To meet the objectives of ensuring compliance and accessibility, many countries have developed national food composition databases tailored to reflect the unique nutritional profiles of locally consumed foods [8,9,10,11]. These databases often contain nutritional information in English, making them suitable for certain applications but limiting their use in multilingual product labels such as English and Arabic. While several techniques have been proposed to automate the process of extracting nutritional information from product label images using deep learning and computer vision methods [2,12,13,14,15,16,17,18], these approaches have predominantly focused on (i) English labels, (ii) those that require a large amount of manually labeled training data, and (iii) those that cannot be be directly accessed and utilized by consumers and online stores in the product label extraction process.

Building on these efforts, the lack of publicly available datasets containing multilingual product labels, particularly in both English and Arabic, remains a significant challenge. Existing food datasets, such as those documented in [19], include vague product names (e.g., “Fruit cake”) without specifying serving sizes and have not been updated since 2011. Another study by Bawajeeh et al. [20] focused on collecting nutrition facts for Arabic food but focused on food dishes rather than product labels. Additionally, datasets such as Aldhirgham et al. [21], which focus on Saudi Arabic food, are neither publicly available nor well structured and unified, further limiting their usability. A recent effort by Buthaina et al. [22] aimed to create a food product label dataset but concentrated only on Omani-branded food, with just 571 images containing English labels, and is not publicly available. Moreover, none of these datasets have been accompanied by algorithms to automate the extraction of nutritional facts. This reliance on manual transcription not only causes human fatigue but also significantly increases the likelihood of errors.

In this study, we address the existing gaps by manually creating a comprehensive dataset of multilingual product labels, including both English and Arabic, to facilitate the extraction of nutritional information and make health-related and logistics decisions. Our empirical analysis evaluates the performance of state-of-the-art large language models (LLMs) in extracting nutritional elements and values from these labels. To overcome the challenges posed by multilingual data and improve extraction accuracy, we applied a series of post-processing techniques, including text restructuring, dictionary-based corrections, and numeral standardization. These techniques significantly enhanced the accuracy of the LLMs, particularly for Arabic labels, with notable improvements in extracting both elements and values compared to the raw outputs. This work can be incorporated into the FDA’s workflow to automate the extraction of food label data, removing the need for manual entry. The extracted information can then be made publicly available to inform consumers or support further research. Additionally, retailers could use this tool to extract data from food label images, enhancing their applications with valuable information that consumers can access when purchasing products.

Our online appendix https://zenodo.org/records/14630762 (accessed on 5 December 2024) includes the full dataset of product labels along with their corresponding nutritional elements and values in both English and Arabic. It also provides the extraction script with the designed prompts, the post-processing script, and a comprehensive analysis of the results.

The rest of the paper is organized as follows. In Section 2, we review the related work. Section 3 provides an overview of our empirical study, followed by the presentation of results and key findings in Section 4. Potential threats to the validity of our study are discussed in Section 5. Finally, we conclude the paper and outline future directions in Section 6.

## 2. Related Work

### 2.1. Food Composition Database

Several efforts have been made to construct Food Composition Databases (FCDBs) to provide comprehensive information on the nutritional content of foods, supporting dietary assessments, and public health. Many of these efforts have been led by national Food and Drug Authorities, which aim to address the specific dietary needs of their populations and focused on local products with labels in their native language such as Greece [9], the United Kingdom [23], the United States [24], France [25], and Belgium [26]. However, they primarily focus on single-language datasets, often English, and are not designed to address the challenges of multilingual product labels needed in regions like Arabic-speaking countries nor experimenting LLMs in their studies.

Currently, only limited efforts have been made to create Arabic food composition datasets. For example, Bawajeeh et al. [20] developed a food database called *myfood24*, which primarily focuses on local food dishes rather than systematically collecting local product labels. Similarly, a recent database created by Buthaina et al. [22] focused on Omani-branded foods but was limited in size, and the product labels did not include both English and Arabic. A similar database created by Habib et al. [27] focused exclusively on food dishes consumed in the United Arab Emirates. A more closely related study focused on Saudi-branded food by Aldhirgham et al. [21], but the dataset is not publicly available. These existing studies relied on manually transcribing nutritional information, a process that is not only labor-intensive but also prone to human error. To address these limitations, we created our own database, which includes product labels in both English and Arabic, transcribed manually to ensure accuracy. Unlike previous efforts, our work goes a step further by aiming to automate the extraction process through a comprehensive empirical evaluation of state-of-the-art large language models applied to the data we collected.

### 2.2. Extracting Information from Food Labels

Research into automated food label analysis has primarily focused on applying optical character recognition (OCR) and various post-processing techniques to extract critical information such as harmful ingredients, allergens, expiration dates, and nutritional values.

For harmful ingredient detection, Kamis et al. [28] applied Tesseract OCR followed by tokenization and lexicon-based filtering to identify risky components from ingredient lists. Parkavi et al. [29] developed a mobile application that extracts food names using OCR and cross-references them with allergen databases, using a matching algorithm to alert users with allergies. Similarly, Akram et al. [7] combined OCR and barcode scanning to classify food as halal, detect allergens, and offer nutritional guidance. Lam et al. [6] used an OCR and augmented reality (AR) approach to scan E-Code labels and brand names, facilitating real-time ingredient validation. Despite these efforts, most methods are rule-based and limited to detecting predefined ingredients, lacking flexibility in handling different wording, multilingual terminology, or layout differences in labels. Despite these efforts, most methods are rule-based and rely on predefined ingredient lists or barcode databases. They often require extensive post-processing to correct OCR errors and are not well suited for multilingual labels or complex visual layouts.

Several works have focused on expiration date recognition, a challenging task due to noise, font variation, and image quality. Gong et al. [4] employed a dual deep neural network architecture for detecting “use by” dates, addressing variability in lighting and packaging design. Grumeza et al. [5] integrated LLaMA2 with EasyOCR, fine-tuning it on cropped expiration date images for better detection accuracy. However, these approaches are typically task-specific and focus on isolated date fields rather than comprehensive nutritional labels.

For diet management and nutrition extraction, Sheela et al. [30] developed NutriGaze, which combines OCR with natural language processing (NLP) to detect sugar-related terms, aiding diabetic users. Kulyukin et al. [17] created a smartphone-based system utilizing Tesseract and GOCR OCR engines to localize and interpret nutrition labels. Nasir et al. [31] used Tesseract OCR to extract daily values (DVs) from nutritional tables. D’Ambrosio et al. [32] proposed Easy Eat, which applies computer vision to parse nutritional composition tables and employs machine learning to recommend healthier alternatives. However, these systems typically assume well-formatted, single-language labels and require extensive post-processing, making them less suitable for bilingual or noisy real-world datasets.

In summary, prior work has addressed specific food label extraction tasks but remains constrained by reliance on OCR alone, predefined rule sets, and limited multilingual support. In contrast, our approach integrates OCR with large language models (LLMs) to enable robust, context-aware extraction from bilingual English–Arabic labels, offering a more scalable and generalizable solution. To the best of our knowledge, this is the first work to apply LLMs for structured nutritional information extraction across languages.

### 2.3. Large Language Models in Food Applications

Several works have demonstrated the potential of ChatGPT-based applications in food and health. For example, these models improve meal personalization [33]. GPT-4 and GPT-4v have shown remarkable capabilities in nutrition and dietary analysis, supporting tasks such as nutrition estimation [34], generating personalized recipes [35], and recognizing food items and portion sizes through multimodal inputs [36]. Additionally, these models facilitate ingredient-level nutritional data creation for food classification and recipe generation [37]. Hua et al. [34] further expanded on this by evaluating twelve leading LLMs, including GPT-4o, Llama3.1, Qwen2, Gemma2, and OpenBioLLM, specifically for their performance in nutrition estimation.

Other prominent approaches involved the use of LLaMA and LLaMA2 to enhance accessibility and cross-modal retrieval. For example, LLaMA2 has been applied to detect expiration dates of food products, providing audio feedback to visually impaired users and supporting tasks like grocery shopping [5]. Additionally, these models have been employed for cross-modal alignment and recipe retrieval, effectively bridging the semantic gap between textual and visual data. Similarly, Song et al. [38] introduced a data-augmented retrieval approach, leveraging LLaMA2 to generate visual descriptions from textual recipes, thereby improving image-to-text alignment and enabling recipe searches based on food images. Alternatively, LLaVA-Chef enhances multimodal reasoning by aligning visual inputs with food-related content generation, generating ingredient lists and cooking instructions directly from food images through multi-stage training on recipe-specific data [39].

Each of these studies highlights the growing potential of LLMs in addressing complex food-related tasks. Each of these studies highlights the growing potential of LLMs in addressing complex food-related tasks. However, prior LLM work has not addressed bilingual nutritional label extraction, particularly for Arabic–English pairs, nor evaluated post-processing for accuracy improvement. Hence, our work bridges this gap by evaluating LLMs for bilingual extraction, addressing challenges such as alternative wording in raw outputs, and introducing post-processing to enhance accuracy.

## 3. Empirical Study

**Figure 1:**
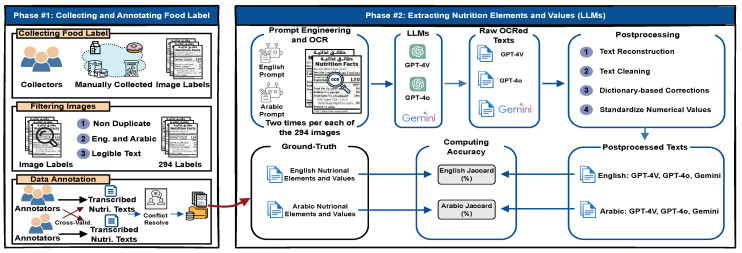
An overview of our study for extracting bilingual nutritional information from food labels using large language models (LLMs) and post-processing techniques.

In this section, we outline the methodology employed in our empirical study, which consists of two primary phases as illustrated in Figure 1: (1) collecting and annotating food labels and (2) extracting nutrition elements and values using large language models (LLMs). During the first phase, we gathered nutritional label images from publicly available sources, carefully filtered the data, and annotated/transcribed the relevant nutritional information. In the second phase, we employed prompt engineering to fine-tune and instruct three widely used LLMs for extracting information from the collected images. We then applied a four-step post-processing procedure to refine the results and evaluated the accuracy of each LLM.

Through this empirical study, we formulated the following five research questions to evaluate the performance of LLMs in extracting nutritional information from bilingual nutritional labels.

***RQ_1_*** 
*How accurate can LLMs extract English elements from nutritional labels with English and Arabic Elements?*
***RQ_2_*** 
*How accurate can LLMs extract English elements and Values from nutritional labels with English and Arabic Elements?*
***RQ_3_*** 
*How accurate can LLMs extract Arabic elements from nutritional labels with English and Arabic Elements?*
***RQ_4_*** 
*How accurate can LLMs extract Arabic elements and values from nutritional labels with English and Arabic Elements?*
***RQ_5_*** 
*Does the language (Arabic vs. English) affect the accuracy of LLMs in extracting elements and values from nutritional labels with English and Arabic elements?*

In the following subsections, we provide a detailed explanation of each step for both phases: (1) collecting food label images, filtering the images, and annotating nutritional data and (2) prompt engineering for nutritional information extraction, post-processing the extracted data, and evaluating the model accuracy.

### 3.1. Collection and Annotating Process

#### 3.1.1. Collecting Food Labels

We began our study by collecting images of nutritional labels that display nutritional information in both English and Arabic. The images were sourced from publicly available databases involved browsing popular publicly accessible online grocery listings and product catalogs commonly used in the Gulf region, covering a variety of food categories including bakery, canned food, sauces and dressings, sweets and snacks, beverages, and dairy products. To ensure consistency and reliability in the data collection process, we established clear criteria for three Software Engineering students tasked with gathering the images.

**Figure 2:**
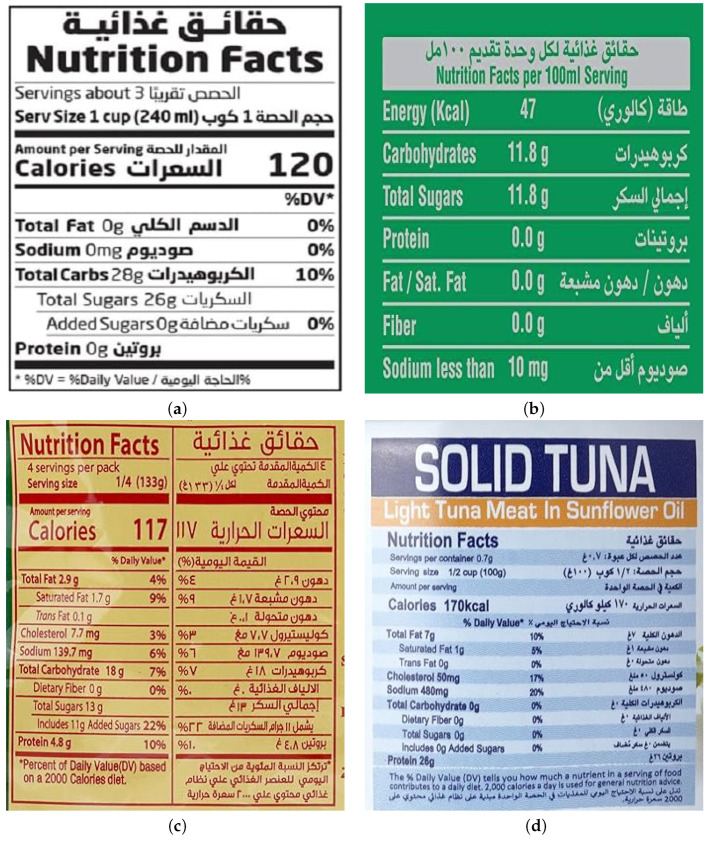
Examples of nutritional labels with varying nutrition fact table structures in both English and Arabic. Labels (**a**,**b**) show standard and clear formats, whereas labels (**c**,**d**) demonstrate alternative layouts and designs, illustrating the diversity in label structures used across different food categories and sources.

Before starting, we defined a set of rules and guidelines to standardize the selection of images, ensuring that the labels were legible and contained both English and Arabic nutritional data. Students were also instructed to seek images with different nutrition fact table structures as nutritional labels vary in format (see Figure 2 for examples of varying label structures).

Additionally, to avoid duplication, each data collector was instructed to collect images from a different public source and category, minimizing the chances of overlap. This process resulted in a total of 350 images.

#### 3.1.2. Filtering Nutrition Label Images

We proceeded with filtering the 350 collected images to ensure data quality by identifying and removing (i) duplicate images, (ii) images that did not contain both English and Arabic text, and (iii) images with illegible nutritional information. This process was carried out by one of the authors and verified by another to ensure consistency.

Duplicates were defined as multiple images displaying the same product label, and, in such cases, only one image was retained. Images with unreadable or blurred text, where the nutritional details could not be clearly read, were removed. Additionally, any image that contained only English or only Arabic text was excluded from the dataset. After applying these filters, 294 images were retained for our empirical study.

#### 3.1.3. Annotating Nutritional Data

During this step, five different annotators were involved to ensure accuracy and consistency. We first divided the dataset (294 images) among three undergraduate students, assigning each annotator 98 images to manually transcribe. They were tasked with transcribing both the nutritional elements and their corresponding values in both English and Arabic. The element names were written exactly as they appeared on the labels (e.g., “Total fat”), followed by the corresponding value (e.g., 16 g). This process led to the complete annotation of 294 images, yielding a total of 588 annotations, with 294 in English and 294 in Arabic.

To further ensure accuracy, two additional annotators re-annotated the entire dataset, with each re-annotating 147 images. A cross-check was then performed to compare their annotations with those of the initial annotators. Any discrepancies, such as typos in the nutritional element names (e.g., “Total Fats” instead of “Total Fat”), misspelled values (e.g., “1.5 g” entered as “15 g”), or extra spaces in the text, were identified and resolved collaboratively. The initial Cohen’s Kappa coefficient was 0.91, which indicated a high agreement between the annotators.

In analyzing the dataset, we observed a range in the number of nutritional elements per image: the minimum was 3, the maximum reached 22, and the average was approximately 10.44 elements per image. These values reflect the range and typical depth of nutritional detail provided across products, offering insight into the standard level of nutritional information captured in food labels.

During our manual analysis of the nutritional information in our dataset, we observed a variation in the number of nutritional elements per image, ranging from a minimum of 3 to a maximum of 22, with an average of approximately 10.44 elements per image. This diversity highlights the varying levels of nutritional detail provided across products, reflecting the variety in the nutritional information captured in food labels within our dataset.

The total number of elements in the dataset was 3160, highlighting the substantial volume of nutritional information analyzed in this study. Table 1 presents the top 5 most frequent nutritional elements found in the dataset, listed in English along with their respective counts. Protein was the most frequently listed element, appearing in 277 labels, followed by Sodium with 267 occurrences, indicating the prominence of these key nutritional components across the collected food products.

### 3.2. Extracting Nutrition Information

#### 3.2.1. Prompt Engineering

To extract nutritional elements and values from our product dataset, we developed specific zero-shot prompts to guide large language models (LLMs) in accurately interpreting and responding to our queries. Nutritional information in this context is defined as a set of element–value pairs that describe the core components listed on food labels (e.g., Calories, Protein, Sodium, and Total Fat) and is presented in both English and Arabic. The goal was to extract these elements and their associated numeric values excluding daily values, ingredient lists, or unrelated textual content. The underlying principle of our approach relies on the multimodal reasoning capabilities of advanced LLMs (e.g., GPT-4o and GPT-4V), which can interpret both textual and visual input. By crafting structured prompts, we instruct the model to simulate how a human would read and interpret a nutrition label table and to output the results in a standardized format. This enables direct comparison with the manually annotated ground truth and simplifies downstream processing. To further ensure the quality of extracted data, we designed prompts that explicitly request the nutrition table content only in the target language (either English or Arabic), and we applied a post-processing pipeline to restructure, clean, and normalize the output for accurate evaluation.

After experimenting with several prompt formulations, we identified the following prompts as the most effective for extracting nutritional information in the desired structured format for both languages:


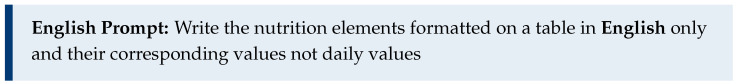



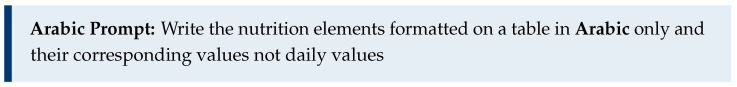


Note that without specifying “only,” the responses occasionally included elements in multiple languages, which was not the intended outcome. Additionally, the model sometimes extracted daily values alongside the primary values, despite the prompt explicitly requesting only the corresponding nutritional values. By emphasizing these constraints in the prompt, we aimed to refine the output to better align with the intended format and content.

#### 3.2.2. Nutritional Information Extraction

To perform the nutritional information extraction, we utilized GPT-4V (https://openai.com/index/gpt-4v-system-card/ (accessed on 5 June 2025)), GPT-4o (https://openai.com/index/hello-gpt-4o/ (accessed on 5 June 2025)) (which support multilingual capabilities), and Gemini (https://gemini.google.com/app (accessed on 5 June 2025)), which are among the state-of-the-art large language models (LLMs). These models were selected for their advanced language support and accuracy, having been proven robust in extracting information from images in previous studies. GPT-4V is based on a modular vision–language architecture, combining a separate vision encoder with a text decoder (GPT-4), which makes it particularly effective at structured OCR and visual text extraction tasks. GPT-4o, on the other hand, introduces a unified architecture that natively handles multiple modalities—including text, image, and audio—enabling more fluid, real-time multimodal reasoning. Gemini incorporates a deep fusion transformer with a Mixture of Experts (MoE) design, allowing dynamic routing across specialized sub-models and supporting large-scale context integration. We used the default temperature setting (1.0) for both GPT-4V and GPT-4o. For max token limits, GPT-4V was configured with 300 tokens and GPT-4o with 1000 tokens in image-based completion tasks. Gemini was used via the gemini-pro-vision API with default parameters.

For each image in our dataset, which contains both English and Arabic nutritional labels, we instructed each LLM with the previously formulated prompts to extract nutritional elements and values. Each model generated responses in separate files: one file for Arabic and another for English. Formally, let $LLM_{Engine\_lang}$ represent the LLM applied on the image to extract text in a specified language. For each LLM engine and language, we denote the extracted output for an image as $OCRed_{img}$, resulting in a set of OCRed files:

$$LLM_{Engine\_lang}=\left\{OCRed_{img1},OCRed_{img2},OCRed_{img3},\ldots ,OCRed_{imgn}\right\}$$

where *Engine* denotes the model (GPT-4V, GPT-4o, or Gemini), and *lang* specifies the language (English or Arabic).

After completing this process, we generated a total of 1764 OCRed files: 294 files for each language (English and Arabic) across the three engines, providing comprehensive coverage of the dataset for our empirical evaluation.

#### 3.2.3. Post-Processing the Raw Nutritional Information

Initially, the extracted nutritional information showed low accuracy across all engines, with Arabic performing the worst. A manual investigation into the results revealed several recurring issues that affected the structured output. To address these, we implemented a post-processing pipeline consisting of four key steps to refine and standardize the raw OCRed data and ensure a robust comparison with the ground truth. These post-processing steps were lightweight, introducing minimal computational overhead relative to LLM inference time, and were necessary to correct structural inconsistencies, resolve variations in terminology, and standardize numeral formats.

**Text Restructuring**: We noted that the OCRed files did not always follow a consistent table structure. In some cases, new columns were added, or two columns were merged, resulting in an unstructured format. To resolve this, we developed a language-specific script to process each file (in both English and Arabic), reformatting it into a standard table structure. This enabled the files to be easily parsed into a dictionary format for further analysis.

**Text Cleaning:** The LLMs occasionally included additional information unrelated to the nutritional elements and values, such as percentage daily values or units within the element names. To address this, we created a script that iterates through each row, checking each column’s content to ensure it contained only relevant information (i.e., the nutritional element name and its corresponding value) and removing any extraneous data.

**Dictionary-Based Corrections:** We observed that the LLMs sometimes used synonyms instead of the exact terms found in the images. For instance, “carbohydrate” was sometimes represented as “total carbs,” “calories” as “energy,” and “protein” as “proteins”. To support consistency in evaluation, we created a dictionary for both English and Arabic nutritional elements that lists all known synonyms for each element, as shown in Table 2. This allows us to standardize terms by converting any synonym found in either the ground truth or the predictions back to its original form. Note that the dictionary was used solely for evaluation to normalize equivalent terms and does not require updates as it does not affect model outputs or deployment. The complete dictionary we created is included in our replication package for reference (https://zenodo.org/records/14630762 (accessed on 5 June 2025)).

**Standardize numerical values:** For Arabic nutrition values, we encountered inconsistencies in numeral formats, with some values extracted as Arabic–Indic numerals and others as standard numerals. To resolve this, we standardized all numerical values to standard numerals, ensuring consistency in numerical representation, which is important for our empirical evaluation.

### 3.3. Evaluation Metrics

To evaluate the accuracy of the large language models (LLMs) and the associated processing steps, we employed the *Jaccard Index*, a well-established metric for measuring the similarity between two sets. The Jaccard Index calculates the ratio of the intersection (true positives) between the predicted and ground-truth sets to the union of these sets, effectively quantifying the proportion of predicted elements that were correctly identified out of all relevant elements in the ground truth. This metric is particularly useful for evaluating our empirical study as it provides a clear measure of accuracy across different types of nutritional information.

For each image in our dataset, we compared the ground-truth English nutritional information to the information predicted by each of the LLMs. Similarly, we computed the Jaccard Index for Arabic nutritional information to evaluate how well the models performed in extracting information in both languages. To further assess the models, we extended the evaluation to include not only the elements alone but also elements paired with their corresponding values, computing the Jaccard Index for English elements and values, as well as Arabic elements and values.

Formally, in the Jaccard Index calculation, we assessed how many items were correctly extracted by the model (**True Positives (TP)**), how many items were incorrectly extracted (**False Positives (FP)**), and how many ground-truth items were missing from the prediction (**False Negatives (FN)**). Let *P* denote the **Predicted set** of items, including all elements or element–value pairs identified by the LLM, and *G* denote the **Ground-Truth set** of items, which includes all correct elements or element–value pairs according to the actual labeled data. The Jaccard Index is computed as the ratio of the number of true positives to the total number of items across both the ground-truth and predicted sets, including the false positives and false negatives.

The formula is expressed as

(1) $$\mathrm{Jaccard}\;\mathrm{Index}=\frac{\left|P\cap G\right|}{\left|P\cap G|+|P-G|+|G-P\right|}$$

where

- |P∩G| represents the number of **True Positives (TPs)** (nutritional information correctly extracted);
- |P−G| represents the **False Positives (FPs)** (nutritional information incorrectly extracted);
- |G−P| represents the **False Negatives (FNs)** (nutritional information missing from the extraction).

## 4. Empirical Results

To report the results, we needed to parse the raw text into a format that allowed for comparison between the extracted elements and values from the LLMs and the ground-truth data. We achieved this by converting the raw text into a dictionary-based format, where each key represented an element name and each corresponding value held the extracted value. The parsing was conducted by identifying the *|* symbol, which served as a delimiter between each element and its value. For value extraction, we checked if a character was a digit and recorded it as the value for the associated element. This method produced dictionaries in the format {element_1_: value_1_, element_2_: value_2_, …, element_n_: value_n_}, which we then compared with the ground truth. Additionally, we addressed a specific inconsistency where the LLM would return *false* when the value was zero. To standardize the output, we replaced any instance of *false* with the numeric value zero.

The following subsections present the results we obtained, which address the corresponding research questions (RQs).

### 4.1. RQ_1_: Extracting English Elements

The initial results for extracting English elements from bilingual nutritional labels, without any pre-processing, revealed varying levels of accuracy across the models. While all models could identify a substantial number of correct elements, there were notable inconsistencies, particularly in the presence of missing and incorrect extractions. GPT-4V, GPT-4o, and Gemini showed some limitations in accuracy without the post-processing step, with GPT-4o achieving the highest initial Jaccard Index of 0.994.

To enhance extraction accuracy, a post-processing step was introduced that involved the use of standardized dictionaries as discussed in Section 3.2.3. These dictionaries mapped extracted element names to their standard forms, resolving inconsistency in element identification. By ensuring consistency in element naming, the models were able to better match the extracted elements with ground-truth labels, resulting in marked improvements across all models. This dictionary-based correction step notably reduced both missing and incorrect element counts, especially for GPT-4o and GPT-4V, which demonstrated strong performance improvements.

After applying the dictionary-based post-processing, the results demonstrated significant enhancements in accuracy across all models, as summarized in Table 3. For instance, GPT-4o’s Jaccard Index increased by 9.2%, showing a substantial reduction in missing and incorrect elements compared to the initial results. GPT-4V also achieved an improved Jaccard Index of 0.992, while Gemini reached 0.956 after processing, reflecting a significant improvement of 18.5%.

Our insights reveal that LLMs often use alternative names for nutritional elements and sometimes deviate from the requested format, which necessitated post-processing to standardize and structure the extracted data. By applying these adjustments, we achieved a more consistent alignment with ground-truth labels. The final results indicate promising accuracy and reliability in extracting English elements from bilingual nutritional labels.


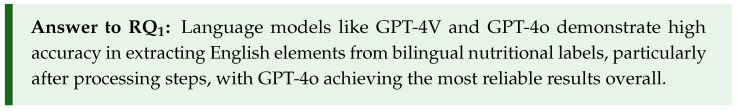


### 4.2. RQ_2_: Extracting English Elements and Values

In this analysis, we compared the full element–value pairs extracted from bilingual nutritional labels. For accuracy, both the element and its associated value had to be correct; an error in either was counted as an incorrect extraction. This approach allowed for a more precise and rigorous evaluation of the models’ ability to extract the full nutritional information accurately.

Beyond the dictionary standardization discussed previously, additional processing steps included standardizing numerical formats and decimal points to ensure consistency across extracted values, as discussed in Section 3.2.3. This refinement helped reduce discrepancies and improved the alignment of extracted data with the ground-truth information.

After processing, the results indicate an improvement in extraction accuracy across all models, as shown in Table 4. GPT-4V and GPT-4o, in particular, demonstrated notable improvements, with Jaccard Indices increasing due to reductions in missing and incorrect values. Overall, this structured approach to processing resulted in more accurate and reliable extraction of English elements and values from nutritional labels.

After processing, the results indicate an improvement in extraction accuracy across all models, as shown in Table 4. For GPT-4V, the percentage of correct extractions increased from 88.0% to 90.1%, with missing values decreasing from 12.0% to 9.9%, and the number of incorrect extractions reduced from 454 to 377. Similarly, GPT-4o showed notable improvements, with correct extractions increasing from 82.5% to 87.3%, missing values decreasing from 17.5% to 12.7%, and incorrect extractions reduced from 663 to 489. Gemini also demonstrated significant enhancements, with correct extractions increasing by 9.4%, missing values decreasing by 9.4% percentage points, and incorrect extractions reduced by 272 instances.

Our insights from the results suggest that accurately extracting both elements and values from nutritional labels requires careful standardization. All LLMs showed improved performance when numerical formats were standardized, reinforcing the importance of the processing in aligning model output with the precise ground-truth data. These adjustments resulted in notably higher accuracy, demonstrating that structured processing enhances the reliability of extracted nutritional information.


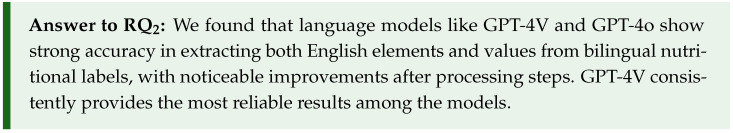


### 4.3. RQ_3_: Extracting Arabic Elements

The task of extracting Arabic elements from bilingual nutritional labels required specific handling to address the challenges of Arabic–Indic numerals commonly found in such labels. Initial results showed a wide range in accuracy across the models, with GPT-4o demonstrating the highest performance among the models before any post-processing.

To improve accuracy, we implemented a processing step to convert Arabic–Indic numerals into standard numerals, facilitating consistency in extracted values as explained in Section 3.2.3. This conversion involved mapping Arabic–Indic numerals to their corresponding standard numerals, ensuring uniformity across the extracted data. This processing step, in addition to standardizing Arabic element names based on a manually created dictionary, enhanced the overall accuracy and reduced discrepancies in numerical data.

As shown in Table 5, the processing steps significantly improved performance across all models. For instance, GPT-4o’s Jaccard Index increased by 79.2%, rising from 19.2% to 98.4%, due to a substantial reduction in both missing elements and incorrect extractions. Similarly, GPT-4V improved by 78.9%, with its Jaccard Index increasing from 17.3% to 96.2%, while Gemini saw a notable improvement of 82.3%, increasing from 8.9% to 91.2%. Notably, GPT-4o achieved the highest highest performance after post-processing.

Our insights from the results indicate that converting Arabic–Indic numerals to standard numerals significantly improved extraction accuracy. This processing step, along with standardizing element names, was crucial in reducing missing and incorrect elements. The improved performance, particularly evident in GPT-4o, highlights the importance of targeted post-processing steps for the precise extraction of Arabic elements in bilingual nutritional labels.


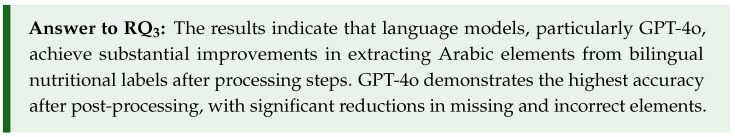


### 4.4. RQ_4_: Extracting Arabic Elements and Values

The extraction of Arabic elements and their associated values from bilingual nutritional labels required the LLMs to accurately identify and pair each element with its correct value. Similar to our evaluation for the English elements–values, we considered both elements and values together, meaning an extraction was counted as correct only if both the element and its corresponding value were accurate.

In addition to standardizing Arabic element names and converting Arabic–Indic numerals to standard numerals, our processing steps included enforcing consistency in numerical formats, particularly decimal points (e.g., “,” changed to “.”). This step helped to minimize variations in extracted values and improved the alignment with the ground-truth data, resolving any discrepancies.

The results, as shown in Table 6, reveal substantial improvements post-processing across all models. GPT-4o demonstrated the greatest gains in accuracy, achieving a notable increase in its Jaccard Index from 0.178 to 0.725, with a significant reduction in missing and incorrect element–value pairs. GPT-4V and Gemini also showed improvements, though to a lesser extent, indicating the continued need for refined pre-processing to handle complex Arabic nutritional labels effectively.

As shown in Table 6, post-processing steps resulted in substantial performance improvements across all models. GPT-4V achieved the most significant gains, with its Jaccard Index increasing by 56%, from 16.5% to 72.5%. This improvement was accompanied by a notable reduction in both missing values (from 74.1% to 20.0%) and incorrect element–value pairs (from 2300 to 546). Similarly, GPT-4o saw a substantial improvement, with its Jaccard Index rising by 53.8%, from 17.8% to 71.6%, reflecting enhanced accuracy and a decrease in missing elements and incorrect pairs. Gemini also showed improvement, with its Jaccard Index increasing by 53.6%, from 11.2% to 64.8%.

To better understand the sources of extraction errors, we performed a qualitative analysis of common failure cases in Arabic outputs. We found that most errors stemmed from OCR misreads of Arabic–Indic numerals, inconsistent use of alternative terminology, and layout ambiguities in label structures (e.g., merged cells or misaligned columns). These issues led to missing or incorrect element–value pairs, particularly before post-processing. Our targeted corrections such as numeral standardization, dictionary-based normalization, and table restructuring—directly addressed these issues and contributed significantly to the observed accuracy.

These insights suggest that extracting both Arabic elements and values accurately requires robust processing to standardize numerals and use of Arabic dictionaries. The improvement in performance, particularly in GPT-4o, highlights the importance of precise processing steps for accurate extraction in bilingual nutritional labels, demonstrating the effectiveness of targeted adjustments for Arabic data in bilingual nutritional labels.


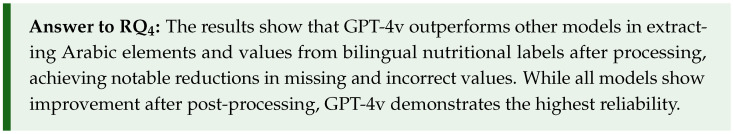


### 4.5. RQ_5_: Language Impact on Extraction Accuracy

**Figure 3:**
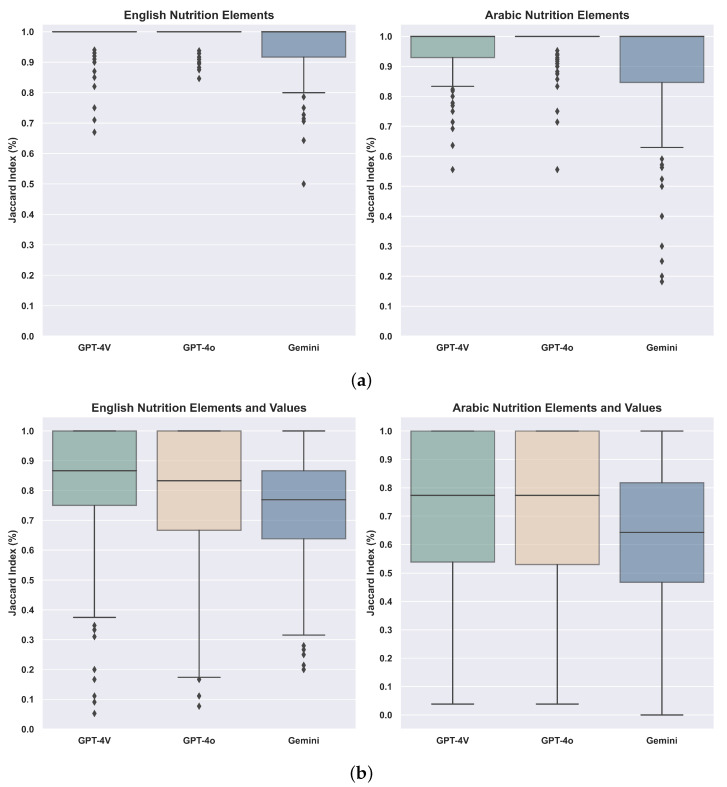
Boxplots showing how well LLMs perform on extracting English and Arabic elements and values. (**a**) Boxplots of LLMs performance in extracting English and Arabic elements. (**b**) Boxplots of LLMs performance in extracting English and Arabic elements with values.

Figure 3 summarizes the results for (i) extracting nutritional elements in English and Arabic and (ii) extracting nutritional elements along with their associated values for both languages. As shown in Figure 3a, GPT-4V and GPT-4o outperformed Gemini in extracting English elements, with GPT-4o demonstrating the best performance for Arabic elements. This highlights the robustness of GPT-4o in handling multilingual data, aligning with its capabilities announced during its release. Figure 3b further illustrates that GPT-4o and GPT-4V performed similarly in extracting English elements and values, achieving a median accuracy of approximately 83–87%, compared to Gemini’s 77%. However, the accuracy for extracting Arabic elements and values declined by around 6–10% for both GPT-4V and GPT-4o and by 13% for Gemini, reflecting the added complexity of handling Arabic text with values. Note that these performance differences can be partially attributed to architectural distinctions. GPT-4V’s modular vision–language design contributes to its strong image-text extraction capabilities, while GPT-4o’s unified multimodal model enables more robust reasoning across noisy layouts and inconsistent formatting. In contrast, Gemini’s lower performance may stem from differences in tokenization or a lack of optimization for Arabic script despite its advanced architecture and extended context handling.


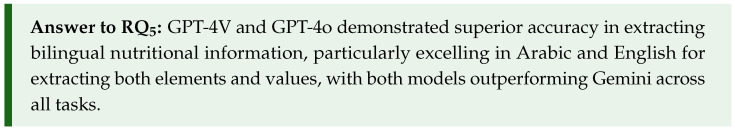


## 5. Threats to Validity

### 5.1. Threats to Validity

Our study faced several key challenges that could influence the reliability of our findings. These challenges are categorized into internal, construct, and external validity concerns, as detailed below.

#### 5.1.1. Internal Validity

Internal validity is influenced by potential errors in manual annotation or transcription of nutritional labels, which could affect the accuracy of the ground truth data despite employing cross-validation. To mitigate this, we implemented a rigorous multi-stage process involving five annotators. The dataset was initially divided among three annotators, with two additional annotators re-annotating and cross-checking the data to identify and resolve discrepancies collaboratively. This process ensured consistency and accuracy, further validated by achieving a high inter-annotator agreement with a Cohen’s Kappa coefficient of 0.91.

#### 5.1.2. Construct Validity

Construct validity concerns arise from the reliance on the Jaccard Index and the specific prompt designs for LLMs, which could impact evaluation consistency. To address this, we ensured that the Jaccard Index was appropriate for evaluating the extracted nutritional information by aligning it with prior studies that have successfully utilized it for similar tasks. Furthermore, we standardized the LLM prompt designs to minimize variability and ensure fair comparisons across models, especially for multilingual and structurally diverse labels.

#### 5.1.3. External Validity

External validity concerns arise due to the dataset’s limited size (294 food labels) and its representativeness as it may not fully capture the diversity of nutritional labels across languages and product types. We mitigated this threat by including labels with various label layouts, ensuring diverse presentations of nutritional information, and incorporating multilingual labels in both English and Arabic. These labels were sourced from different product categories, such as beverages, snacks, and dairy products, to account for variability in design and content.

## 6. Conclusions

In this study, we evaluated the capabilities of large language models (LLMs) for extracting nutritional information from bilingual labels, focusing on multilingual content, mixed-language formatting, and diverse label designs. Using a dataset of 294 bilingual labels, we demonstrated that LLMs, particularly GPT-4o, achieved reliable performance in extracting both English and Arabic nutritional information, outperforming GPT-4V and Gemini. This highlights the adaptability of LLMs in processing multilingual data without the need for domain-specific training datasets.

As part of future work, we aim to develop few-shot prompt strategies to minimize the reliance on post-processing by encouraging more structured outputs directly from the models. We also plan to explore the extraction of nutritional data from Arabic-only labels, which may present new challenges such as dialectal variation, brand-specific phrasing, or informal terminology. Addressing these challenges will likely require improved synonym handling, expanded language normalization, and possibly domain-adapted or fine-tuned LLMs tailored for Arabic food labeling contexts.

## Figures and Tables

**Table 1:** Top 5 most frequent nutritional elements in English and Arabic.

Rank	Element Name	Count
1	Protein	277
2	Sodium	267
3	Total Fat	256
4	Calories	244
5	Cholesterol	241

**Table 2:** Examples of key nutritional elements and their alternative names from our manually created dictionary.

Key Element Name	Alternative Names
Protein	Proteins, total protein
Carbohydrate	Carbohydrates, carbohydrates total, total carbs
Saturated Fat	of which saturated fat, saturated fats, sat fat, saturated

**Table 3:** The results of extracting English elements from nutritional labels before and after processing steps using GPT-4V, GPT-4o, and Gemini.

LLM	Before Processing				After Processing			
	**%Correct**	**%Missing**	**#Incorrect**	**Jacc.**	**%Correct**	**%Missing**	**#Incorrect**	**Jacc.**
GPT-4V	0.961 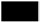	0.039 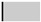	94	0.946	0.992 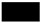	0.008 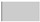	8	0.992
GPT-4o	0.937 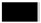	0.063 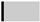	198	0.902	0.997 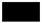	0.003 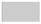	15	0.994
Gemini	0.835 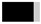	0.165 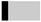	382	0.771	0.961 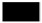	0.039 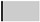	33	0.956

**Table 4:** The results of extracting English elements and values from nutritional labels before and after processing steps using GPT-4V, GPT-4o, and Gemini.

LLM	Before Processing				After Processing			
	**%Correct**	**%Missing**	**#Incorrect**	**Jacc.**	**%Correct**	**%Missing**	**#Incorrect**	**Jacc.**
GPT-4V	0.880 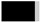	0.120 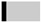	454	0.810	0.901 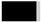	0.099 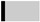	377	0.836
GPT-4o	0.825 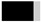	0.175 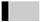	663	0.735	0.873 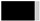	0.127 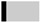	489	0.795
Gemini	0.738 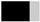	0.262 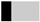	776	0.633	0.832 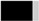	0.168 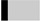	504	0.745

**Table 5:** The results of extracting Arabic elements from nutritional labels before and after processing steps using GPT-4V, GPT-4o, and Gemini.

LLM	Before Processing				After Processing			
	**%Correct**	**%Missing**	**#Incorrect**	**Jacc.**	**%Correct**	**%Missing**	**#Incorrect**	**Jacc.**
GPT-4V	0.286 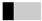	0.714 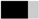	2299	0.173	0.967 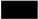	0.033 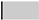	32	0.962
GPT-4o	0.313 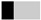	0.687 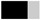	2263	0.192	0.985 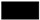	0.015 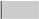	9	0.984
Gemini	0.161 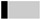	0.839 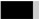	2547	0.089	0.930 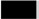	0.070 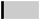	107	0.912

**Table 6:** The results of extracting Arabic elements and values from nutritional labels before and after processing steps using GPT-4V, GPT-4o, and Gemini.

LLM	Before Processing				After Processing			
	**%Correct**	**%Missing**	**#Incorrect**	**Jacc.**	**%Correct**	**%Missing**	**#Incorrect**	**Jacc.**
GPT-4V	0.259 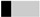	0.741 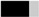	2300	0.165	0.800 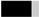	0.200 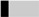	546	0.725
GPT-4o	0.278 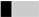	0.722 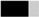	2281	0.178	0.796 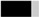	0.204 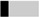	592	0.716
Gemini	0.190 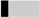	0.810 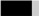	2408	0.112	0.740 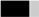	0.260 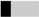	694	0.648

## Data Availability

The data presented in this study are openly available at https://zenodo.org/records/14630762 (accessed on 5 June 2025).
